# Wie oft braucht es eine Thoraxdrainageneinlage beim Thoraxtrauma des schwerer Verletzten – und wann mehr? Grundlagen eines Schweizer Traumazentrums zur Planung von Ressourcen und Ausbildungskapazitäten

**DOI:** 10.1007/s00104-020-01292-7

**Published:** 2020-10-09

**Authors:** Stephanie Walkner, Felix Amsler, Thomas Gross

**Affiliations:** 1 Klinik für Chirurgie, Kantonsspital Aarau, Tellstr. 1, 5001 Aarau, Schweiz; 2 Amsler Consulting, Gundeldingerrain 111, 4059 Basel, Schweiz; 3 Klinik für Traumatologie, Kantonsspital Aarau, Tellstr. 1, 5001 Aarau, Schweiz; 4 Klinik für Orthopädie und Traumatologie des Bewegungsapparates, Kantonsspital Baselland, 4101 Bruderholz, Schweiz

**Keywords:** Facharztausbildung, Notfallchirurgie, Thoraxverletzung, Mindesteingriffszahl, Traumazentrum, Specialist medical training, Emergency surgery, Thoracic injury, Minimum intervention number, Trauma center

## Abstract

**Hintergrund und Fragestellung:**

Im Hinblick auf den Ressourcen- und Ausbildungsbedarf eines Schweizer Traumazentrums wollten wir wissen, wie häufig in der Schwerverletztenversorgung relevante Thoraxverletzungen auftreten und wie oft eine thoraxchirurgische Spezialversorgung notwendig ist.

**Material und Methoden:**

Retrospektive Analyse aller von 2010 bis 2017 notfallmäßig mit einem Mindesttrauma-NISS (New Injury Severity Score) ≥8 betreuten Patienten bez. relevanter Thoraxverletzung (Abbreviated Injury Scale [AIS] Thorax [ohne Brustwirbelverletzungen] ≥2).

**Ergebnisse:**

In der 7‑jährigen Beobachtungsperiode wurden 2839 Verletzte mit einem NISS ≥8 notfallmäßig behandelt. Davon erlitten 791 Patienten (27,9 %) eine relevante Thoraxverletzung. Von diesen bedurften 27,1 % (*n* = 215) eines Thoraxeingriffes, der in 86,5 % (*n* = 186) allein einer Thoraxdrainage und in 13,5 % (*n* = 29) einem weitergehenden Eingriff entsprach. Bei 19 der Thoraxverletzten musste darüber hinaus ein Thoraxchirurg gerufen werden, davon 4‑mal sofort und 4‑mal innerhalb von 24 h. Die in unserem Haus im Mittel 30 notfallmäßigen Thoraxdrainageneinlagen pro Jahr ermöglichten 1 bis 2 Eingriffe pro in Ausbildung stehendem Chirurg.

**Diskussion:**

Im Beobachtungszeitraum benötigten nur 1 % aller relevant Thoraxverletzten eine über eine Pleuradrainage hinausgehende, notfallmäßige thoraxchirurgische Versorgung. Aufgrund dieser geringen Rate erscheint aus Effizienz- wie Kostengründen ein thoraxchirurgischer Präsenzdienst an einem derartigen Traumazentrum nicht angezeigt. Die Fähigkeit der Thoraxdrainageneinlage muss allerdings in der chirurgischen Ausbildung entsprechend vermittelt werden. Die gemäß Facharztausbildungskatalogen notwendigen Mindesteingriffszahlen sollten angesichts der erhobenen Fallzahlen gut erfüllbar sein.

**Zusatzmaterial online:**

Zusätzliche Informationen sind in der Onlineversion dieses Artikels (10.1007/s00104-020-01292-7) als Supplementary Material enthalten.

## Abstract (en)

**Background and objective:**

With respect to the resource and training requirements of a Swiss trauma center, we wanted to know how frequently relevant thoracic injuries occur and how often specialized thoracic surgery is needed.

**Material and methods:**

Retrospective analysis of all severely injured patients with a new injury severity score (NISS) ≥8 from 2010–2017 with respect to relevant thoracic injuries (abbreviated injury scale, AIS thorax without thoracic vertebral injuries ≥2).

**Results:**

In the 7‑year observational period 2839 patients with NISS ≥8 were treated as an emergency. Of these 791 (27.9%) suffered a relevant injury in the thoracic region and 27.1% (*n* = 215) of them required a thoracic intervention, which in 86.5% (*n* = 186) corresponded to a thoracic drainage only and in 13.5% (*n* = 29) to an extended intervention. In 19 cases following relevant thoracic injury, a thoracic surgeon was also required, 4 times immediately and 4 times within 24 h of hospital arrival. On average, 30 emergency thoracic drainage insertions per year corresponded to 1–2 interventions per surgeon in training.

**Conclusion:**

In the observational period only 1% of all relevant thoracic injuries required emergency thoracic surgical care beyond a pleural drainage. Given this low rate, for efficiency and cost reasons a thoracic surgery on-call service appears to be appropriate and compulsory attendance is not needed; however, the capability to insert a thoracic drainage must be appropriately assured by surgical training. In view of the number of cases observed per trainee, the minimum number of interventions required according to specialty training regulations should be well achievable.

Die Thoraxdrainageneinlage ist ein obligatorischer Eingriff nationaler und internationaler chirurgischer Facharzt-Curricula [13, 14] sowie gleichzeitig einer der essenziellen Notfalleingriffe im Rahmen der Schwerverletztenversorgung [1, 2, 6, 12, 19]. Leitlinien wie Übersichtsarbeiten fordern, dass jeder Chirurg diesen Eingriff beherrscht [16]. In der Literatur fehlen allerdings genaue Alltagsangaben zum tatsächlichem Ressourcen- und Ausbildungsbedarf mitteleuropäischer Traumazentren, auch in Abgrenzung zur thoraxchirurgischen Spezialversorgung.

## Hintergrund

Das Thoraxtrauma zählt weltweit zu den häufigsten Unfallfolgen und ist nach Schädel-Hirn- und Abdominalverletzungen die dritthäufigste Todesursache weltweit [8, 11]. Abhängig von der Schwere der Verletzung bzw. Behandlung und Komorbidität der Patienten beträgt die Mortalität bis zu 60 % [4, 11]. Der mit dem Thoraxtrauma einhergehende klinische Aufwand ist sehr ressourcenintensiv [11]. Ungeachtet der möglichen gravierenden Folgen einer Brustkorbverletzung wird selbst für Polytraumapatienten mit relevantem stumpfem Thoraxtrauma eine konservative Behandlungsrate (inkl. Thoraxdrainageneinlage) von 50–80 % angegeben [4, 5, 8, 16], d. h. mehrheitlich reicht chirurgisch, neben einer möglichst adäquaten Schmerz- und Atemtherapie, eine Drainageneinlage [4, 7, 8]. Diese erfolgt meist notfallmäßig, teilweise bereits präklinisch [8]. Notfallmäßig notwendige Thorakotomien werden, je nach untersuchter Schwerverletztenkohorte, in etwa 3–10 % [5, 8, 9] angegeben.

Da kaum Angaben zu Häufigkeit und Konsequenzen der Behandlung relevanter Thoraxverletzungen an einem mitteleuropäischen Zentrum vorliegen, führten wir diese Analyse mehrjährig prospektiv-konsekutiv erhobener Daten an einem der 12 Schweizer Traumazentren durch. Dabei ergaben sich folgende zwei Hauptfragestellungen:

1. Inwieweit wird, insbesondere im Rahmen der Notfallversorgung beim Verdacht auf eine Schwerverletzung, routinemäßig ein Thoraxchirurg benötigt und rechtfertigen die Fallzahlen bzw. Ergebnisse einen 24-stündigen thoraxchirurgischen Präsenzdienst?
2. Ist angesichts der vorhandenen Daten die seitens Fachgesellschaften eingeforderte Mindesteingriffszahl an Thoraxdrainagen pro auszubildendem Chirurg realistisch?

## Methoden

Die in einem Studienregister von 2010 bis 2017 prospektiv-konsekutiv erfassten Angaben eines Traumazentrums wurden retrospektiv analysiert. Die Auswertung erfolgte als Teil eines von der zuständigen Ethikkommission genehmigten Versorgungsforschungsprojektes. Das Ausbildungsspital ist eines von 12 Zentrumskrankenhäusern, welches gemäß gesetzlicher Regelung in der Schweiz für die Schwerverletztenversorgung im Rahmen der hochspezialisierten Medizin (HSM; [10]) zugelassen ist. Das Zentrum umfasst ein Einzugsgebiet von etwa 2000 km^2^ mit ca. 750.000 Einwohnern und 400 bis 500 Unfallschockraumeinsätzen pro Jahr. Die Schockraumversorgung [7] bzw. Behandlung beim Verdacht auf eine Schwerverletzung erfolgt gemäß internationalem Standard, wobei mindestens alle chirurgisch-traumatologischen Schockraumdiensthabenden (inkl. Traumaleader) einen ATLS(Advanced Trauma Life Support)©-Provider-Kurs absolvierten. Gemäß den Schweizer Vorgaben für ein HSM-Traumazentrum ist u. a. ein chirurgisch-traumatologisches, anästhesiologisches, radiologisches, neurochirurgisches oder Notfallpflegedienstteam im Sinne eines Präsenzdienstes 24 h pro Tag vor Ort. Als Level-2-Zentrum besitzt das Krankenhaus keine Herzchirurgie, und Spezialdienste wie die Thoraxchirurgie gewährleisten einen Pikettdienst mit Notfallanfahrtszeiten von ca. 30–60 min.

### Studiendesign

Die Ausgangskohorte umfasste alle notfallmäßig (innerhalb von 24 h nach dem Unfall) versorgten Verletzten mit einer erlittenen Mindesttraumaschwere NISS (New Injury Severity Score) ≥8. In Anlehnung an andere Arbeiten [8] definierten wir eine Verletzung der Thoraxregion, unabhängig von einer etwaigen BWS(Brustwirbelsäule)-Verletzung, mit einem AIS (Abbreviated Injury Scale) $\geq$2 als relevantes Trauma. Diese Definition wurde gewählt, um im Kontext dieser spezifizierten Arbeit zur Thoraxeingriffsnotwendigkeit erlittene BWS-Verletzungen von der Thorax-AIS-Berechnung auszuschließen und damit Verfälschungen in der Assoziation von erlittener Thoraxverletzungsschwere und daraus folgenden Thoraxeingriffen bezogen auf die Fragestellung zu vermeiden. Thoraxeingriffe wurden folgendermaßen kategorisiert:

- alleinige Thoraxdrainageeinlage,
- Thorakoskopie/-tomie,
- nichtspezifisch thoraxchirurgisch durchgeführte Thoraxwandeingriffe bzw. den Thoraxraum (mit-)betreffende Laparatomieeingriffe.

Angesichts der Studienziele wurden für diese Auswertung nur notfallmäßige, nicht aber perioperativ routinemäßig eingelegte Thoraxdrainageneinlagen berücksichtigt. Die im Schwerverletztenmanagement notfallmäßig erfolgten Thoraxdrainagen wurden vom chirurgisch-traumatologischen Dienstteam durchgeführt. Für alle notfallmäßigen Thorakoskopien bzw. Thorakotomien wurde hingegen ein Thoraxchirurg hinzugerufen, (semi-)elektiv wurden diese Eingriffe allein vom Thoraxchirurgieteam durchgeführt. Alle übrigen Eingriffe am Thorax wurden durch die jeweiligen Dienstteams der Chirurgie-Traumatologie (z. B. Stichkanalrevision Brustwand) bzw. Viszeralchirurgie (z. B. Laparatomie mit Zwerchfellnaht) oder interventionellen Radiologie (Embolisation) durchgeführt.

Der Zeitpunkt eines Eingriffes wurde kategorisiert nach (a) *notfallmäßig, sofort *im Anschluss an die Schockraum(SR)-Phase vor Verlegung in den Operationssaal, die Intensivstation (IPS) oder auf Station; (b) *innerhalb von 24* *h* oder (c) *>24* *h* nach Spitaleintritt und ob im Falle einer Thorakotomie eine Brustkorbblutstillung ausreichte. Ebenfalls wurde erhoben, ob darüber hinaus spezifische thoraxchirurgische Operationen (an Lunge, Bronchus/Bronchien oder Herz) notwendig waren. Bei sämtlichen Patienten des Studienkollektives wurde überprüft, ob sie innerhalb eines Jahres aufgrund eines thoraxchirurgischen Eingriffes infolge der erlittenen Verletzung rehospitalisiert werden mussten.

Die standardisierte Datenregistrierung erfolgte durch eine nicht in die Patientenbehandlung involvierte „study nurse“ und umfasste detaillierte Angaben zu Demographie, Trauma, Behandlung und Kurzzeitoutcome der Patienten, analog der Eingabe im TraumaRegisterDGU® (http://www.traumaregister-dgu.de) und unter Verwendung etablierter Skalen und Scores gemäß früherer Zitation: [10] Abbreviated Injury Scale, AIS; (New) Injury Severity Score, (N)ISS; Glasgow Coma Scale, GCS; Age and Systolic Blood Pressure, GAP; altersunabhängiger und altersadjustierter Charlson-Score; Leistungserfassung von Pflegeleistungen gesamthaft, LEP (http://www.lep.ch); Revised Injury Severity Score 2, RISC2; penetrierendes vs. nichtpenetrierendem Trauma. Als Mehrfachverletzung wurde eine Verletzungskombination mit je AIS >0 in mindestens zwei Körperregionen bezeichnet, als Polytrauma musste eine derartige Verletzungskombination zudem eine Gesamtmindesttraumaschwere im ISS >16 erreichen.

### Analyse und Statistik

Die Kennwerte wurden mit Mittelwert und Standardabweichung (SD) oder Anzahl und Prozent dargestellt. Für Gruppenvergleiche wurden χ^2^-Tests für nominale Daten, Mann-Whitney-U-Test für ordinale und T‑Test für intervallskalierte Variablen angewendet. Um die Stärke der Zusammenhänge zu operationalisieren, wurden die Determinationskoeffizienten r^2^ (entspricht der erklärten Varianz) angegeben. Für die multivariate Auswertung wurde für jeden Vergleich eine block- und schrittweise logistische Regression durchgeführt. In allen Tests wurden dieselben Variablen mit dem Einschlusskriterium *p* < 0,05 einbezogen:

- Block 1, demografische und unfallbezogene Daten: Alter, Geschlecht, altersunabhängiger Charlson-Score, Unfallmechanismus Energie hoch, Unfallmechanismus penetrierend, Transportart Helikopter, Versorgung sekundär, erster systolischer Blutdruck, erste Sauerstoffsättigung, erster GCS, GAP.
- Block 2, verletzungsbezogene Daten: RISC 2, AIS 1–6, AIS Thorax (ohne BWS).
- Block 3, versorgungsbezogene Daten: Intubation im SR, Intubation überhaupt, Blut- oder Hämostasetherapie SR/IPS erhalten, IPS-Aufenthalt, Tage auf der IPS, Hospitalisationsdauer, LEP.
- Block 4, Outcomevariablen: GOS <5 (mindestens, nicht gut erholt), Rehabilitationsaufenthalt nach Austritt, Verstorben im Krankenhaus. (Da sich diese Outcomevariablen in keinem Fall als signifikant erwiesen, wurden sie in den Multivariattabellen nicht aufgeführt.)

Das Signifikanzniveau wurde auf *p* < 0,05 festgelegt. Die Datenanalyse erfolgte mit SPSS 26.

## Ergebnisse

**Figure 1:**
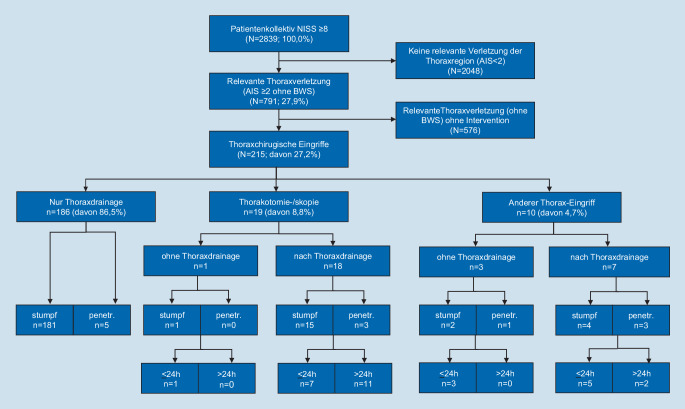

Insgesamt wurden in der 7‑jährigen Beobachtungsperiode 2839 Verletzte mit einem signifikanten Trauma (NISS ≥8) notfallmäßig behandelt, wovon 983 Patienten (34,6 %) eine Verletzung der Thoraxregion (AIS >0) erlitten hatten. Das Studienkollektiv umfasste diejenigen 791 Patienten mit einer *relevanten Thoraxverletzung *(AIS ≥2, ohne BWS-Verletzungen; Abb. 1). Diese erwiesen sich in der univariaten Analyse gegenüber den übrigen untersuchten Verletzten als häufiger männlich, erfuhren häufiger eine hohe Unfallenergie, waren schwerer verletzt bzw. häufiger polytraumatisiert und blieben bei zugleich höherem Pflegeaufwand (LEP) im Mittel länger im Spital (alle *p* < 0,001). Es fand sich kein Unterschied bez. Alter oder Spitalletalität (s. Supplementary Material Tab. A).

Bei 27,1 % (*n* = 215) dieser relevant Brustkorbverletzten war (mindestens) *ein Thoraxeingriff,* d. h. zumindest die Einlage einer Thoraxdrainage notwendig, davon 3‑mal präklinisch. In der multivariaten Regressionsanalyse erklärte vor allem eine zunehmende Thoraxverletzungsschwere (AIS) sowie die Tatsache eines penetrierenden Traumas die Notwendigkeit zur Durchführung eines Thoraxeingriffes (zusammen 22 % der erklärten Varianz). Das univariat schlechtere Outcome bei den Patienten nach Thoraxeingriff (inkl. Thoraxdrainage), mit häufigerem Rehabilitationsbedarf und GOS-Status <5 (s. Supplementary Material Tab. B), wurde in der multivariaten Analyse nicht bestätigt (Tab. 1). Als Indikation für einen Thoraxeingriff fand sich in 85,6 % (*n* = 184) ein diagnostizierter Hämato- und/oder Pneumothorax. 89,3 % (*n* = 192) der Betroffenen wiesen Rippenfrakturen auf. In 86,5 % der Fälle (*n* = 186) bestand die notwendige Thoraxintervention allein aus dem Einlegen einer Drainage (bis maximal 3 Drainagen pro Fall).

**Table 1:** 

Block	Schritt	Variablen	B	*p*	OR	95 %-CI			Gesamt		Verbesserung	
						Unterer	Oberer		*p*	R^2^	*p*	R^2^
Demografie und Unfall	1	Penetrierend	2,28	<0,001	9,816	2,90	33,29	9,816	<0,001	0,03	–	–
Verletzung	2	AIS Thorax (ohne BWS)	1,06	<0,001	2,873	2,10	3,93	2,873	<0,001	0,17	<0,001	0,14
	3	AIS6 Weichteile	−0,46	0,007	0,631	0,45	0,88	0,631	<0,001	0,18	<0,012	0,01
Versorgung	4	LEP Total	0,00005	<0,001	1,00005	1,00003	1,00007	1,000	<0,001	0,25	<0,001	0,07
	5	Intubiert	0,64	0,002	1,90	1,26	2,85	1,895	<0,001	0,27	0,002	0,01
–	–	Constant	−4,64	<0,001	0,01	–	–	–	–	–	–	–

*AIS* Abbreviated Injury Scale, *B* Regressionskoeffizient B,* BWS *Brustwirbelsäule, *Gesamt* Gesamtmodell, *OR* Odds Ratio = EXP (B), *LEP* Leistungserfassung von Pflegeleistungen,* R*^*2*^ Nagelkerke R^2^, *Verbesserung* Verbesserung des Modells pro Schritt, *95* *%-CI* 95- %-Konfidenzintervall

In 29 Fällen (alle AIS Thorax >2) erfolgte *ein weitergehender Eingriff am Thorax*: In 10 Fällen wurde dieser vom nichtthoraxchirurgischen Dienstteam durchgeführt (Embolisation, lokale Thoraxwandrevision oder Laparatomie mit Brustkorbbeteiligung, z. B. bei Zwerchfellruptur) und in 19 Fällen unter Involvierung eines Thoraxchirurgen (Thorakoskopien bzw. -tomien). Sechszehn dieser über eine Thoraxdrainage hinausgehenden Thoraxeingriffe erfolgten innerhalb von 24 h nach Spitaleintritt. Tabelle C im Supplementary Material stellt im univariaten Vergleich die Charakteristika der 186 Verletzten mit *ausschließlicher Thoraxdrainage* den 29 Patienten gegenüber, bei denen (noch) ein anderer Thoraxeingriff notwendig war. Im multivariaten Vergleich zeichneten sich diese durch ein häufiger penetrierendes Trauma (12 % erklärte Varianz), eine höhere Komorbidität (Charlson-Score, 6 %) sowie eine schwerere Thoraxverletzung (6 %, zusammen 24 % der Varianz) aus (Tab. 2). Das Outcome unterschied sich weder im univariaten (s. Supplementary Material Tab. C) noch im multivariaten Vergleich.

**Table 2:** 

Block	Schritt	Variablen	B	*p*	OR	95 %-CI		Gesamt		Verbesserung	
						Unterer	Oberer	*p*	R^2^	*p*	R^2^
Demografie und Unfall	1	Penetrierend	3,04	<0,001	20,91	4,68	93,6	<0,001	0,12	–	–
	2	Charlson-Score	0,34	0,044	1,41	1,01	1,96	<0,001	0,18	0,006	0,06
	3	Weiblich	−1,60	0,059	0,20	0,04	1,07	<0,001	0,23	0,010	0,05
Verletzung	4	AIS Thorax (ohne BWS)	0,93	0,002	2,54	1,41	4,57	<0,001	0,29	0,003	0,06
	5	AIS Extremitäten	−0,55	0,004	0,58	0,40	0,84	<0,001	0,34	0,009	0,05
Versorgung	6	LEP Total	0,00004	0,026	1,00004	1,00001	1,00007	<0,001	0,37	0,031	0,03
–	–	Konstant	−5,25	<0,001	0,01	–	–	–	–	–	–

*AIS* Abbreviated Injury Scale, *B* Regressionskoeffizient B, *BWS *Brustwirbelsäule, *Gesamt* Gesamtmodell, *LEP* Leistungserfassung von Pflegeleistungen,* OR* Odds Ratio = EXP (B), *R*^*2*^ Nagelkerke R^2^, *Verbesserung* Verbesserung des Modells pro Schritt, *95* *%-CI* 95 %-Konfidenzintervall

Ein entsprechendes Bild ergab sich für die Spezifizierung, wann unter den 791 relevant Thoraxverletzten die Notwendigkeit zum Rufen eines Thoraxchirurgen, zur Durchführung einer Thorakotomie oder -skopie (*n* = 19, s. Supplementary Material Tab. D) bestand (zusammen 27 % der Varianz für die 3 genannten Variablen; s. Supplementary Material Tab. E) bzw. wann im Falle eines notwendigen Thoraxeingriffes (*n* = 215) dieser thoraxchirurgisch durchgeführt wurde (s. Supplementary Material Tab. F). Von den 19 thoraxchirurgischen Operationen erfolgte der Eingriff 8‑mal innerhalb von 24 h und davon 4‑mal notfallmäßig sofort im Anschluss an die Schockraumphase. Acht der 11 späteren thoraxchirurgischen Eingriffe fanden während derselben Hospitalisation und 3 in einer Zweithospitalisation statt (2 via Thorakotomie mit offener Hämatomausräumung und Dekortikation bzw. Pleurektomie sowie 1 als thorakoskopische Ergussausräumung mit Frühdekortikation). Somit war von gesamthaft 215 Patienten, welche eines Thoraxeingriffes bedurften, bei 1,9 % (*n* = 4) der sofortige und bei weiteren 1,9 % (*n* = 4) innerhalb von 24 h nach Eintreffen des Patienten der Beizug eines Thoraxchirurgen notwendig, bis auf einen Fall einer Tracheal- bzw. Hauptbronchusruptur immer unter vorgängiger Thoraxdrainageneinlage.

Fünf Polytraumapatienten mit schweren thorakalen Verletzungen (AIS ≥3, ohne BWS: 3‑mal AIS 5 und 2‑mal AIS 3) verstarben bei Eintritt bzw. im Schockraum: Alle erhielten eine Thoraxdrainage und verstarben aufgrund ihrer Kombinationsverletzungen so rasch unter Reanimation (3-mal bei u. a. gleichzeitigem schwerem Schädel-Hirn-Traum, 2‑mal bei u. a. gleichzeitigem schwerem Abdominal- bzw. Beckentrauma), dass kein operativer Eingriff mehr durchgeführt werden konnte.

Insgesamt fanden sich im Studienkollektiv 12 Fälle mit *penetrierendem Trauma*, welche alle einen Thoraxeingriff erhielten: In 5 Fällen nur eine Thoraxdrainage, bei 3 Patienten eine Thoraxwandrevision nach vorgängiger Thoraxdrainageneinlage, 1‑mal allein eine Thoraxwandrevision sowie 3‑mal eine Thorakotomie/-skopie nach vorgängiger Thoraxdrainageneinlage. Bis auf eine Thorakotomie erfolgten alle diese Eingriffe bei penetrierendem Trauma notfallmäßig.

## Diskussion

Die vorliegende Studie untersucht, unseres Wissens erstmals für ein europäisches Traumazentrum, an über 2800 konsekutiv erfassten signifikant Verletzten die Häufigkeit und chirurgische Behandlungsnotwendigkeit eines dabei erlittenen relevanten Thoraxtraumas. Bisherige Arbeiten zur Thematik beziehen sich meist auf Subkohorten, z. B. allein Schwerverletzte bzw. Polytraumatisierte [4, 8, 11] oder unter Ausschluss von z. B. Schädel-Hirn-Verletzungen [4]. Es fanden sich dabei folgende zwei Hauptergebnisse:

### Rund-um-die-Uhr-Vorhaltung eines Thoraxchirurgen

*Erstens* betrug der Anteil der relevanten Thoraxverletzten 28 %, wovon 27 % eines chirurgischen Eingriffes bedurften, welcher allerdings fast immer allein einer Thoraxdrainageneinlage entsprach. Bei 2,4 % aller Thoraxverletzten wurde ein Thoraxchirurg (hinzu)gerufen, davon nur 4‑mal in 7 Jahren (0,5 %) notfallmäßig, sofort nach Spitaleintritt; dies, obwohl der thoraxchirurgische Pikettdienst prinzipiell immer zu einer Thorakotomie gerufen wurde, sogar wenn diese bereits von einem anderen Fachchirurgen begonnen wurde und potenziell auch zu Ende hätte geführt werden können. Selbst bei den (wenigen) penetrierenden Thoraxtraumata war ein thoraxchirurgischer Einsatz nur in jedem 4. Fall erforderlich, auch wenn alle Fälle situationsentsprechend notfallmäßig revidiert wurden.

Angesichts der im Rahmen der erwarteten risikoadaptierten Letalität liegenden Spitalmortalität, multivariat nicht verschiedenem Outcome zwischen den Vergleichsgruppen sowie der zur Literatur [8, 17] passenden Vergleichsrate des Verhältnisses von Thoraxdrainage zu Thorakotomie/-skopie von über 12:1 rechtfertigen die Ergebnisse für das untersuchte Kohortenspektrum keine „Rund-um-die-Uhr-Vorhaltung“ eines thoraxchirurgischen Dienstarztes im Spital, auch wenn dessen Einsatzspektrum selbstverständlich mehr als nur unfallchirurgische Indikationen umfasst. Ein Piketthintergrunddienst mit 30- bis 60-minütiger Anfahrtszeit, wie bei uns praktiziert, genügt unseres Erachtens zumindest für ein „Level-2“-Traumazentrum. Trotz der fortschreitenden Spezialisierung erscheint, auch aus Effizienz- und Kostengründen und ohne Versorgungsqualitätsverlust, der Routineeinsatz eines entsprechend ausgebildeten „Generalisten“ diesbezüglich gerechtfertigt [3].

Im untersuchten Kollektiv fand sich kein Hinweis auf eine mangelhafte Ausführung der Thoraxdrainageneinlage seitens (auszubildenden) Dienst- (Allgemein- bzw. Unfall‑)Chirurgen, welche eine thoraxchirurgische Reinterventionsnotwendigkeit aufgrund einer Fehleinlage bedingt hätte. Dies deckt sich mit internationalen Angaben zum Unterschied der Komplikationshäufigkeit nach Thoraxdrainageneinlage je nach operierender Fachdisziplin (s. unten; [18]). Detaillierte Fallobservationen bez. Qualität der Drainageneinlage respektive zur Notwendigkeit z. B. von Umpositionierungen anhand angefertigter Kontrollröntgenuntersuchungen etc. konnten allerdings nicht durchgeführt bzw. systematisch erfasst werden. Fallzahlen wie Charakteristik und Outcome unserer Kohorte erscheinen vergleichbar mit anderen mitteleuropäischen Zentren [2, 7, 10, 17]. In Anbetracht der geringen Zahl behandelter penetrierender Thoraxtraumata [4] und der diesbezüglich bekannt hohen Rate an resultierenden Thorakotomien (bei öfter komplexen Thoraxorganverletzungen; [20]) ist für diese spezielle Untergruppe an Brustkorbverletzungen allerdings eine in dubio großzügige Alarmierung eines Thoraxchirurgen, wenn nicht sogar die primäre Zuweisung in ein „Level-1“-Traumazentrum (inkl. Herz- und Thoraxchirurgie), empfehlenswert.

### Mindesteingriffszahlen für Auszubildende

*Zweitens *wurden in der untersuchten Zentrums- bzw. Ausbildungsklinik aus unfallchirurgischer Notfallsicht über 7 Jahre 215 Thoraxdrainagen eingelegt. Dies entspricht 30 Eingriffen pro Jahr. Bei einem Assistentenpool von gleichzeitig 24 Ärzten in Ausbildung bedeutet dies im Durchschnitt 1 bis 2 Thoraxdrainageneinlagen pro Auszubildenden. Wenn man nichtunfallchirurgische Indikationen mitberücksichtigt, dürfte ein Assistenzarzt über 6 Jahre sicherlich auf 12 bis 15 derartige Notfalleingriffe an einer A1-Klinik mit voller Ausbildungsbefugnis für Viszeral‑, Unfall- sowie Neurochirurgie kommen. Hinzu kommen noch intraoperative Drainageneinlagen im Rahmen der elektiven Thorax‑, Viszeral- oder Wirbelsäulenchirurgie sowie die an unserer Klinik für alle im Traumaschockraummanagement tätigen allgemein- und unfallchirurgischen Dienstärzte obligate ATLS-Simulationsschulung.

Diesen Fallzahlen stehen die seitens der nationalen chirurgischen Fachgesellschaften eingeforderten Mindesteingriffszahlen von 10 Drainageneingriffen in der deutschen Facharztausbildung, je zum Unfall- wie Viszeralchirurgen [14], bzw. 15 Eingriffen für den Schweizer [13] wie österreichischen Allgemein- und Viszeralchirurgen [15] gegenüber. Aus der Perspektive eines Schweizer Traumazentrums erscheint somit die seitens Fachgesellschaften eingeforderte Mindesteingriffszahl an Thoraxdrainagen pro auszubildendem Chirurg im Rahmen der chirurgischen Ausbildung als realistisch. Es dürften jedoch deutlich weniger solcher Eingriffe an kleineren Kliniken stattfinden bzw. die Expertise für derartige, gerade auch in der Peripherie notwendige Notfalleingriffe muss zumeist im Zentrum erworben werden. Zudem gilt es, sich die sinkenden Fallzahlen pro Auszubildenden aufgrund stetig größer werdender Dienstteams im Rahmen vollzogener Arbeitszeitreduktionen zu vergegenwärtigen [3]. Der Ausbildungs- bzw. Trainingsbedarf seitens als Notärzte tätiger anderer Facharztrichtungen sowie sich neu etablierende Spezialdisziplinen, wie der „emergency physician“ oder „practice provider“ [3], müssen ebenfalls vor dem Hintergrund eines nicht geringer werdenden Qualitätsanspruches auch im Notfallmanagement betrachtet werden. In der Literatur finden sich disziplinenabhängige Komplikationsraten nach Drainageneinlage von 6–7 % bei Thorax- bzw. Allgemeinchirurgen, im Vergleich zu 13 % bei Internisten und 23 % bei Notärzten (23 %; [18]).

### Limitationen

Das retrospektive Monocenterdesign, wenn auch prospektiv erfasster Daten, ist eine der Limitationen dieser Studie. Das Krankenhaus ist allerdings bez. Schwerverletztenversorgung immerhin das viertgrößte von 12 Traumazentren der Schweiz (interne Daten Swiss Trauma Registry, http://www.swisstraumaboard.ch) und aufgrund des 7 Jahre umfassenden Untersuchungszeitraumes ergaben sich unter 2839 signifikant Verletzten des Ausgangskollektivs doch immerhin 791 relevant am Thorax Verletzte, mit einem mittlerer Verletzungsschweregrad AIS 3. Angesichts der wenigen penetrierenden Traumata (bei zugleich fehlender Kardiochirurgie im Hause) können die Resultate gut auf vergleichbar strukturierte Kliniken, nicht ohne Weiteres jedoch z. B. auf urbane „Level-1“-Traumazentren mit häufiger zu behandelnden Schuss- oder Stichverletzungen übertragen werden [20].

Trotz durchgeführter Multivariatanalysen lassen sich angesichts resultierender Varianzangaben von 20–30 % keine therapeutischen Schlussfolgerungen zur spezifischen Operationsindikation von Thoraxdrainage wie Thorakotomie oder dem Rufen eines thoraxchirurgischen Facharztes ziehen. Die dargestellten Analysen beschränken sich, mit Ausnahme der untersuchten Rehospitalisationsnotwendigkeit, auf die erhobenen Spitaldaten. Darüber hinausreichende Langzeitdaten wurden nicht erhoben. Allerdings ist deren spezifische Aussagekraft angesichts der zu erwartenden Kofaktoren bei u. a. 90 % Mehrfachverletzten im Kollektiv sowie der fehlenden Signifikanz der Spitaloutcomeparameter in der Multivariatanalyse als sehr fraglich einzuordnen.

## Fazit für die Praxis

- Bei jedem vierten relevant Brustkorbverletzten war mindestens ein Thoraxeingriff notwendig, welcher sich aber in fast 9 von 10 Fällen auf eine Thoraxdrainageneinlage beschränkte.
- Pro Jahr musste im untersuchten Level-2-Traumazentrum bei relevanter Thoraxverletzung maximal 1‑ bis 2‑mal innerhalb von 24 h nach Spitaleintritt ein Thoraxchirurg gerufen werden.
- Angesichts dieser Ergebnisse erscheint ein thoraxchirurgischer Hintergrunddienst für ein derartiges mitteleuropäisches Traumazentrum ausreichend und
- die für die chirurgischen Facharztausbildungskataloge notwendigen Fallzahlen bez. der Thoraxdrainageneinlage seitens auszubildender Assistenzärzte stellen sich für ein solches Traumazentrum aktuell als gut erfüllbar dar.

## Caption Electronic Supplementary Material


