# Supplementary material for: How often is a chest tube needed following thoracic trauma in the severely injured—and when is more needed? Data from a Swiss trauma center for planning of resources and surgical training
Source: Chirurg. 2020 Oct 9;92(8):721–8. [Article in German] doi: 10.1007/s00104-020-01292-7 (PMC8324590; doi:10.1007/s00104-020-01292-7)
Supplement: Supplementary file 1 [file 104_2020_1292_MOESM1_ESM.docx]

**Suppl. Tabelle A Deskriptiver Vergleich Ausgangskohorte (NISS≥8):**

**Relevante Thoraxverletzung (AIS≥2, ohne BWS) erlitten ja/ nein**

| AIS Thorax (ohne BWS) ≥2 | Total (N=2839) | nein (N=2048) | ja (N=791) |  |  |
| --- | --- | --- | --- | --- | --- |
|  | Mean +SD | Mean +SD | Mean +SD | R^2^ | P |
| Alter bei Unfall | 55,5 (+/-23,8) | 55,5 (+/-24,9) | 55,5 (+/-20,9) | 0,00 | 0,308 |
| 1. syst. Blutdruck | 140,2 (+/-27,9) | 141,6 (+/-27,3) | 136,4 (+/-29) | 0,01 | <0,001 |
| 1. Sauerstoffsättigung | 95,7 (+/-5,6) | 96,1 (+/-4,6) | 94,4 (+/-7,5) | 0,02 | <0,001 |
| 1. GCS | 13,2 (+/-3,4) | 13,2 (+/-3,4) | 13,1 (+/-3,6) | 0,00 | 0,674 |
| ISS | 14,6 (+/-8,3) | 12,7 (+/-6,6) | 19,4 (+/-10,1) | 0,13 | <0,001 |
| NISS | 19,4 (+/-10,8) | 18 (+/-10,1) | 23 (+/-11,6) | 0,04 | <0,001 |
| GAP | 20,3 (+/-4) | 20,3 (+/-4) | 20,2 (+/-4,2) | 0,00 | 0,772 |
| RISC2 (%) | 9,12 (+/-18,32) | 8,99 (+/-17,6) | 9,43 (+/-20,07) | 0,00 | 0,825 |
| Age unadjusted Charlson Score | 0,61 (+/-1,27) | 0,63 (+/-1,3) | 0,55 (+/-1,19) | 0,00 | 0,170 |
| AIS1 Kopf/Hals | 2,01 (+/-1,66) | 2,22 (+/-1,67) | 1,46 (+/-1,52) | 0,04 | <0,001 |
| AIS2 Gesicht | 0,35 (+/-0,78) | 0,36 (+/-0,8) | 0,31 (+/-0,73) | 0,00 | 0,280 |
| AIS3 Thorax | 0,96 (+/-1,4) | 0,18 (+/-0,6) | 2,98 (+/-0,65) | 0,81 | <0,001 |
| AIS4 Abdomen | 0,44 (+/-0,97) | 0,3 (+/-0,83) | 0,78 (+/-1,2) | 0,05 | <0,001 |
| AIS5 Extremitäten | 1,12 (+/-1,29) | 1,04 (+/-1,28) | 1,35 (+/-1,27) | 0,01 | <0,001 |
| AIS6 Weichteile | 0,4 (+/-0,56) | 0,38 (+/-0,56) | 0,45 (+/-0,55) | 0,00 | <0,001 |
| AIS Schädel/Hirn | 1,92 (+/-1,69) | 2,13 (+/-1,7) | 1,37 (+/-1,51) | 0,04 | <0,001 |
| AIS Thorax (ohne BWS) | 0,85 (+/-1,36) | 0,03 (+/-0,18) | 2,96 (+/-0,63) | 0,93 | <0,001 |
| AIS BWS | 0,25 (+/-0,72) | 0,16 (+/-0,59) | 0,49 (+/-0,95) | 0,04 | <0,001 |
| AIS WS (HWS, BWS, LWS) | 0,55 (+/-1,02) | 0,44 (+/-0,94) | 0,84 (+/-1,15) | 0,03 | <0,001 |
| LEP Total | 4906 (+/-6557) | 4247 (+/-5120) | 6611 (+/-9083) | 0,03 | <0,001 |
| Tage IPS (inkl. 0) | 1,87 (+/-4,63) | 1,34 (+/-3,5) | 3,24 (+/-6,53) | 0,03 | <0,001 |
| Tage Intubation | 3,49 (+/-5,24) | 2,66 (+/-4,07) | 4,86 (+/-6,5) | 0,04 | <0,001 |
| Hospitalisationstage | 10,1 (+/-10,1) | 9,5 (+/-9,2) | 11,6 (+/-12) | 0,01 | <0,001 |
|  | n (%) | n (%) | n (%) | R^2^ | P |
| Geschlecht weiblich | 791 (33,7%) | 754 (36,8%) | 203 (25,7%) | 0,01 | <0,001 |
| Unfallmechanismus Energie hoch | 1270 (45,2%) | 743 (36,7%) | 527 (66,9%) | 0,07 | <0,001 |
| Unfallmechanismus penetrierend | 48 (1,7%) | 32 (1,6%) | 16 (2%) | 0,00 | 0,394 |
| Transport Helikopter | 323 (11,4%) | 172 (8,4%) | 151 (19,1%) | 0,02 | <0,001 |
| Versorgung sekundär | 690 (24,3%) | 569 (27,8%) | 121 (15,3%) | 0,02 | <0,001 |
| ISS ≥16 | 1057 (37,2%) | 595 (29,1%) | 462 (58,4%) | 0,07 | <0,001 |
| Mehrfachverletzung | 1930 (68%) | 1206 (58,9%) | 724 (91,5%) | 0,10 | <0,001 |
| Polytrauma (ISS >16 & 2 AIS-Regionen >0) | 827 (29,1%) | 372 (18,2%) | 455 (57,5%) | 0,15 | <0,001 |
| HSM (ISS ≥20 (Kinder ≥16) oder AIS Schädel/Hirn≥3) | 1418 (49,9%) | 1082 (52,8%) | 336 (42,5%) | 0,01 | <0,001 |
| Maximaler AIS >3 | 692 (24,4%) | 486 (23,7%) | 206 (26%) | 0,00 | 0,198 |
| AIS3 Thorax >2 | 672 (23,7%) | 22 (1,1%) | 650 (82,2%) | 0,73 | <0,001 |
| AIS4 Abdomen >2 | 151 (5,3%) | 79 (3,9%) | 72 (9,1%) | 0,01 | <0,001 |
| AIS Thorax (ohne BWS) >2 | 647 (22,8%) | 0 (0%) | 647 (81,8%) | 0,76 | <0,001 |
| AIS BWS >2 | 42 (1,5%) | 22 (1,1%) | 20 (2,5%) | 0,00 | 0,004 |
| AIS WS (HWS, BWS, LWS) >2 | 137 (4,8%) | 96 (4,7%) | 41 (5,2%) | 0,00 | 0,581 |
| Klin. Notfallintervention (Not-Op, DGU) | 214 (7,5%) | 134 (6,5%) | 80 (10,1%) | 0,00 | 0,001 |
| Chirurg. Not-Eingriff inkl. Präklinik (Tx.drain., Not-OP) | 319 (11,2%) | 135 (6,6%) | 184 (23,3%) | 0,06 | <0,001 |
| Chirurg. Not-Eingriff inkl. Präklinik (Tx.drain., Not-OP) oder Damage Control | 472 (16,6%) | 255 (12,5%) | 217 (27,4%) | 0,03 | <0,001 |
| Thoraxdrainage (Präklinik oder SR) | 129 (4,5%) | 1 (0%) | 128 (16,2%) | 0,12 | <0,001 |
| Blut- oder Hämostasetherapie SR/IPS erhalten | 538 (19%) | 328 (16%) | 210 (26,5%) | 0,01 | <0,001 |
| DGU IPS Aufenthalt (aus Zeiten) | 1226 (43,2%) | 845 (41,3%) | 381 (48,2%) | 0,00 | 0,001 |
| Intubiert Präklinik oder SR | 460 (16,2%) | 300 (14,6%) | 160 (20,2%) | 0,00 | <0,001 |
| Intubiert (Präklinik bis IPS, ohne OP) | 642 (22,6%) | 401 (19,6%) | 241 (30,5%) | 0,01 | <0,001 |
| OP AIS3 Thorax | 177 (6,2%) | 33 (1,6%) | 144 (18,2%) | 0,09 | <0,001 |
| OP AIS4 Abdomen | 141 (5%) | 74 (3,6%) | 67 (8,5%) | 0,01 | <0,001 |
| OP AIS Thorax (ohne BWS) | 108 (3,8%) | 1 (0%) | 107 (13,5%) | 0,10 | <0,001 |
| OP AIS WS (HWS, BWS, LWS) | 174 (6,1%) | 97 (4,7%) | 77 (9,7%) | 0,01 | <0,001 |
| OP durchgeführt | 1420 (50%) | 1032 (50,4%) | 388 (49,1%) | 0,00 | 0,523 |
| OP ohne Schädel | 1163 (41%) | 808 (39,5%) | 355 (44,9%) | 0,00 | 0,008 |
| GOS<5 (mind. nicht gut erholt) | 801 (28,9%) | 582 (29,2%) | 219 (28,2%) | 0,00 | 0,601 |
| Reha nach Austritt | 673 (23,7%) | 454 (22,2%) | 219 (27,7%) | 0,00 | 0,002 |
| Verstorben im Krankenhaus | 274 (9,7%) | 189 (9,2%) | 85 (10,7%) | 0,00 | 0,220 |

Die Stärke des statistischen Zusammenhangs und die Signifikanz (t bzw. p) wird durch die Intensität der jeweils verwendeten Farbe veranschaulicht. GCS, Glasgow Coma Scale; (N)ISS, (N)Injury Severity Score; BWS, Brustwirbelsäule; GAP, GCS, Alter und systolischer Blutdruck, RISC2, Revised Injury Severity Classification 2; AIS, Abbreviated Injury Scale;, LEP, LeistungsErfassung von Pflegeleistungen gesamthaft; GOS, Glasgow Outcome Score.

**Suppl. Tabelle B Deskriptiver Vergleich der Studienpopulation mit vs. ohne Thorax-Eingriff (inkl. Thoraxdrainageneinlage)**

| Thorax-Eingriff (ohne BWS) | Total (N=791) | nein (N=576) | ja (N=215) |  |  |
| --- | --- | --- | --- | --- | --- |
|  | Mean +SD | Mean +SD | Mean +SD | R^2^ | P |
| Alter bei Unfall | 55,5 (+/-20,9) | 55,7 (+/-21,4) | 55 (+/-19,3) | 0,00 | 0,533 |
| 1. syst. Blutdruck | 136,4 (+/-28,9) | 138,4 (+/-27,8) | 131,2 (+/-31,4) | 0,01 | <0,001 |
| 1. Sauerstoffsättigung | 94,4 (+/-7,5) | 95 (+/-6,6) | 92,8 (+/-9,5) | 0,02 | <0,001 |
| 1. GCS | 13,1 (+/-3,6) | 13,2 (+/-3,4) | 12,7 (+/-4) | 0,00 | 0,364 |
| ISS | 19,4 (+/-10,1) | 17,8 (+/-8,6) | 23,5 (+/-12,3) | 0,06 | <0,001 |
| NISS | 23 (+/-11,6) | 20,8 (+/-9,9) | 28,9 (+/-13,7) | 0,09 | <0,001 |
| GAP | 20,2 (+/-4,2) | 20,3 (+/-4,1) | 19,8 (+/-4,3) | 0,00 | 0,049 |
| RISC2 (%) | 9,43 (+/-20,07) | 8,28 (+/-18,97) | 12,53 (+/-22,51) | 0,00 | <0,001 |
| Age unadjusted Charlson Score | 0,55 (+/-1,19) | 0,57 (+/-1,2) | 0,52 (+/-1,15) | 0,00 | 0,678 |
| AIS1 Kopf/Hals | 1,46 (+/-1,52) | 1,46 (+/-1,48) | 1,43 (+/-1,61) | 0,00 | 0,458 |
| AIS2 Gesicht | 0,31 (+/-0,73) | 0,32 (+/-0,72) | 0,28 (+/-0,74) | 0,00 | 0,154 |
| AIS3 Thorax | 2,98 (+/-0,65) | 2,85 (+/-0,53) | 3,32 (+/-0,79) | 0,10 | <0,001 |
| AIS4 Abdomen | 0,78 (+/-1,2) | 0,68 (+/-1,11) | 1,05 (+/-1,39) | 0,02 | 0,001 |
| AIS5 Extremitäten | 1,35 (+/-1,27) | 1,29 (+/-1,23) | 1,53 (+/-1,36) | 0,01 | 0,015 |
| AIS6 Weichteile | 0,45 (+/-0,55) | 0,48 (+/-0,55) | 0,39 (+/-0,56) | 0,01 | 0,013 |
| AIS Schädel/Hirn | 1,37 (+/-1,51) | 1,38 (+/-1,48) | 1,35 (+/-1,61) | 0,00 | 0,421 |
| AIS Thorax (ohne BWS) | 2,96 (+/-0,63) | 2,84 (+/-0,51) | 3,3 (+/-0,78) | 0,11 | <0,001 |
| AIS BWS | 0,49 (+/-0,95) | 0,47 (+/-0,92) | 0,56 (+/-1,02) | 0,00 | 0,283 |
| AIS WS (HWS, BWS, LWS) | 0,84 (+/-1,15) | 0,81 (+/-1,1) | 0,91 (+/-1,26) | 0,00 | 0,547 |
| LEP Total | 6611 (+/-9083) | 4975 (+/-6710) | 10979 (+/-12526) | 0,09 | <0,001 |
| Tage IPS (inkl. 0) | 3,24 (+/-6,53) | 2,04 (+/-4,03) | 6,46 (+/-9,98) | 0,09 | <0,001 |
| Tage Intubation | 4,86 (+/-6,5) | 3,21 (+/-3,85) | 6,54 (+/-8,07) | 0,07 | 0,010 |
| Hospitalisationstage | 11,6 (+/-12) | 10 (+/-10,5) | 16 (+/-14,4) | 0,05 | <0,001 |
|  | n (%) | n (%) | n (%) | R^2^ | P |
| Geschlecht weiblich | 215 (25,7%) | 159 (27,6%) | 44 (20,5%) | 0,01 | 0,041 |
| Unfallmechanismus Energie hoch | 527 (66,9%) | 388 (67,6%) | 139 (65%) | 0,00 | 0,484 |
| Unfallmechanismus penetrierend | 16 (2%) | 4 (0,7%) | 12 (5,6%) | 0,02 | <0,001 |
| Transport Helikopter | 151 (19,1%) | 99 (17,2%) | 52 (24,2%) | 0,01 | 0,026 |
| Versorgung sekundär | 121 (15,3%) | 85 (14,8%) | 36 (16,7%) | 0,00 | 0,490 |
| ISS ≥16 | 462 (58,4%) | 311 (54%) | 151 (70,2%) | 0,02 | <0,001 |
| Mehrfachverletzung | 724 (91,5%) | 535 (92,9%) | 189 (87,9%) | 0,01 | 0,025 |
| Polytrauma (ISS >16 & 2 AIS-Regionen >0) | 455 (57,5%) | 310 (53,8%) | 145 (67,4%) | 0,02 | 0,001 |
| HSM (ISS ≥20 (Kinder ≥16) oder AIS Schädel/Hirn≥3) | 336 (42,5%) | 213 (37%) | 123 (57,2%) | 0,03 | <0,001 |
| Maximaler AIS >3 | 206 (26%) | 113 (19,6%) | 93 (43,3%) | 0,06 | <0,001 |
| AIS3 Thorax >2 | 650 (82,2%) | 454 (78,8%) | 196 (91,2%) | 0,02 | <0,001 |
| AIS4 Abdomen >2 | 72 (9,1%) | 38 (6,6%) | 34 (15,8%) | 0,02 | <0,001 |
| AIS Thorax (ohne BWS) >2 | 647 (81,8%) | 451 (78,3%) | 196 (91,2%) | 0,02 | <0,001 |
| AIS BWS >2 | 20 (2,5%) | 13 (2,3%) | 7 (3,3%) | 0,00 | 0,427 |
| AIS WS (HWS, BWS, LWS) >2 | 41 (5,2%) | 25 (4,3%) | 16 (7,4%) | 0,00 | 0,080 |
| Klin. Notfallintervention (Not-Op, DGU) | 80 (10,1%) | 41 (7,1%) | 39 (18,1%) | 0,03 | <0,001 |
| Chirurg. Not-Eingriff inkl. Präklinik (Tx.drain., Not-OP) | 184 (23,3%) | 41 (7,1%) | 143 (66,5%) | 0,39 | <0,001 |
| Chirurg. Not-Eingriff inkl. Präklinik (Tx.drain., Not-OP) oder Damage Control | 217 (27,4%) | 69 (12%) | 148 (68,8%) | 0,32 | <0,001 |
| Thoraxdrainage (Präklinik oder SR) | 128 (16,2%) | 0 (0%) | 128 (59,5%) | 0,52 | <0,001 |
| Blut- oder Hämostasetherapie SR/IPS erhalten | 210 (26,5%) | 113 (19,6%) | 97 (45,1%) | 0,07 | <0,001 |
| DGU IPS Aufenthalt (aus Zeiten) | 381 (48,2%) | 239 (41,5%) | 142 (66%) | 0,05 | <0,001 |
| Intubiert Präklinik oder SR | 160 (20,2%) | 92 (16%) | 68 (31,6%) | 0,03 | <0,001 |
| Intubiert (Präklinik bis IPS, ohne OP) | 241 (30,5%) | 128 (22,2%) | 113 (52,6%) | 0,09 | <0,001 |
| OP AIS3 Thorax | 144 (18,2%) | 27 (4,7%) | 117 (54,4%) | 0,33 | <0,001 |
| OP AIS4 Abdomen | 67 (8,5%) | 33 (5,7%) | 34 (15,8%) | 0,03 | <0,001 |
| OP AIS Thorax (ohne BWS) | 107 (13,5%) | 0 (0%) | 107 (49,8%) | 0,42 | <0,001 |
| OP AIS WS (HWS, BWS, LWS) | 77 (9,7%) | 43 (7,5%) | 34 (15,8%) | 0,02 | <0,001 |
| OP durchgeführt | 388 (49,1%) | 228 (39,6%) | 160 (74,4%) | 0,10 | <0,001 |
| OP ohne Schädel | 355 (44,9%) | 202 (35,1%) | 153 (71,2%) | 0,10 | <0,001 |
| GOS<5 (mind. nicht gut erholt) | 219 (28,2%) | 147 (25,9%) | 72 (34,3%) | 0,01 | 0,021 |
| Reha nach Austritt | 219 (27,7%) | 135 (23,4%) | 84 (39,1%) | 0,02 | <0,001 |
| Verstorben im Krankenhaus | 85 (10,7%) | 58 (10,1%) | 27 (12,6%) | 0,00 | 0,315 |

Die Stärke des statistischen Zusammenhangs und die Signifikanz (t bzw. p) wird durch die Intensität der jeweils verwendeten Farbe veranschaulicht. GCS, Glasgow Coma Scale; (N)ISS, (N)Injury Severity Score; GAP, GCS, Alter und systolischer Blutdruck, RISC2, Revised Injury Severity Classification 2; AIS, Abbreviated Injury Scale;, LEP, LeistungsErfassung von Pflegeleistungen gesamthaft; GOS, Glasgow Outcome Score.

**Suppl. Tabelle C Deskriptiver Vergleich der Patienten mit Thorax-Eingriff: Allein mit Thoraxdrainage vs. mit (späterem) anderem Thoraxeingriff versorgt**

| Thoraxeingriff (N=215) | Nur Thoraxdrainage (N=186) | Anderer Thoraxeingriff (N=29) |  |  |
| --- | --- | --- | --- | --- |
|  | Mean +SD | Mean +SD | R^2^ | P |
| Alter bei Unfall | 55,4 (+/-19) | 52,5 (+/-21,5) | 0,00 | 0,555 |
| 1. syst. Blutdruck | 132 (+/-30,1) | 125,8 (+/-38,4) | 0,00 | 0,166 |
| 1. Sauerstoffsättigung | 92,8 (+/-9,8) | 92,4 (+/-7,6) | 0,00 | 0,584 |
| 1. GCS | 12,5 (+/-4,1) | 13,8 (+/-2,3) | 0,01 | 0,818 |
| ISS | 23,5 (+/-12,5) | 23 (+/-11,6) | 0,00 | 0,898 |
| NISS | 28,8 (+/-13,7) | 29,4 (+/-13,9) | 0,00 | 0,826 |
| GAP | 19,7 (+/-4,5) | 20,4 (+/-2,6) | 0,00 | 0,779 |
| RISC2 (%) | 12,6 (+/-23,39) | 12,12 (+/-16,05) | 0,00 | 0,059 |
| Age unadjusted Charlson Score | 0,44 (+/-0,96) | 1,03 (+/-1,9) | 0,03 | 0,156 |
| AIS1 Kopf/Hals | 1,52 (+/-1,64) | 0,9 (+/-1,32) | 0,02 | 0,050 |
| AIS2 Gesicht | 0,3 (+/-0,77) | 0,17 (+/-0,54) | 0,00 | 0,440 |
| AIS3 Thorax | 3,26 (+/-0,78) | 3,72 (+/-0,75) | 0,04 | 0,001 |
| AIS4 Abdomen | 1,03 (+/-1,38) | 1,17 (+/-1,49) | 0,00 | 0,593 |
| AIS5 Extremitäten | 1,63 (+/-1,33) | 0,83 (+/-1,37) | 0,04 | 0,004 |
| AIS6 Weichteile | 0,39 (+/-0,55) | 0,38 (+/-0,62) | 0,00 | 0,755 |
| AIS Schädel/Hirn | 1,44 (+/-1,64) | 0,79 (+/-1,29) | 0,02 | 0,044 |
| AIS Thorax (ohne BWS) | 3,24 (+/-0,76) | 3,72 (+/-0,75) | 0,05 | 0,001 |
| AIS BWS | 0,59 (+/-1,05) | 0,34 (+/-0,77) | 0,01 | 0,263 |
| AIS WS (HWS, BWS, LWS) | 0,95 (+/-1,3) | 0,62 (+/-0,94) | 0,01 | 0,250 |
| LEP Total | 10414 (+/-12268) | 14603 (+/-13745) | 0,01 | 0,058 |
| Tage IPS (inkl. 0) | 6,03 (+/-9,74) | 9,2 (+/-11,21) | 0,01 | 0,109 |
| Tage Intubation | 5,58 (+/-7,12) | 12,56 (+/-11,03) | 0,09 | 0,006 |
| Hospitalisationstage | 15,63 (+/-14,65) | 18,04 (+/-12,67) | 0,00 | 0,196 |
|  | n (%) | n (%) | R^2^ | P |
| Geschlecht weiblich | 42 (22,6%) | 2 (6,9%) | 0,02 | 0,052 |
| Unfallmechanismus Energie hoch | 123 (66,5%) | 16 (55,2%) | 0,01 | 0,237 |
| Unfallmechanismus penetrierend | 5 (2,7%) | 7 (24,1%) | 0,10 | <0,001 |
| Transport Helikopter | 42 (22,6%) | 10 (34,5%) | 0,01 | 0,165 |
| Versorgung sekundär | 30 (16,1%) | 6 (20,7%) | 0,00 | 0,543 |
| ISS ≥16 | 130 (69,9%) | 21 (72,4%) | 0,00 | 0,784 |
| Mehrfachverletzung | 168 (90,3%) | 21 (72,4%) | 0,04 | 0,006 |
| Polytrauma (ISS >16 & 2 AIS-Regionen >0) | 128 (68,8%) | 17 (58,6%) | 0,01 | 0,278 |
| HSM (ISS ≥20 (Kinder ≥16) oder AIS Schädel/Hirn≥3) | 108 (58,1%) | 15 (51,7%) | 0,00 | 0,523 |
| Maximaler AIS >3 | 76 (40,9%) | 17 (58,6%) | 0,01 | 0,073 |
| AIS3 Thorax >2 | 167 (89,8%) | 29 (100%) | 0,02 | 0,072 |
| AIS4 Abdomen >2 | 26 (14%) | 8 (27,6%) | 0,02 | 0,062 |
| AIS Thorax (ohne BWS) >2 | 167 (89,8%) | 29 (100%) | 0,02 | 0,072 |
| AIS BWS >2 | 7 (3,8%) | 0 (0%) | 0,01 | 0,290 |
| AIS WS (HWS, BWS, LWS) >2 | 16 (8,6%) | 0 (0%) | 0,01 | 0,102 |
| Klin. Notfallintervention (Not-Op, DGU) | 31 (16,7%) | 8 (27,6%) | 0,01 | 0,157 |
| Chirurg. Not-Eingriff inkl. Präklinik (Tx.drain., Not-OP) | 121 (65,1%) | 22 (75,9%) | 0,01 | 0,253 |
| Chirurg. Not-Eingriff inkl. Präklinik (Tx.drain., Not-OP) oder Damage Control | 126 (67,7%) | 22 (75,9%) | 0,00 | 0,382 |
| Thoraxdrainage (Präklinik oder SR) | 109 (58,6%) | 19 (65,5%) | 0,00 | 0,483 |
| Blut- oder Hämostasetherapie SR/IPS erhalten | 80 (43%) | 17 (58,6%) | 0,01 | 0,117 |
| DGU IPS Aufenthalt (aus Zeiten) | 118 (63,4%) | 24 (82,8%) | 0,02 | 0,041 |
| Intubiert Präklinik oder SR | 56 (30,1%) | 12 (41,4%) | 0,01 | 0,227 |
| Intubiert (Präklinik bis IPS, ohne OP) | 98 (52,7%) | 15 (51,7%) | 0,00 | 0,923 |
| OP AIS3 Thorax | 93 (50%) | 24 (82,8%) | 0,05 | 0,001 |
| OP AIS4 Abdomen | 29 (15,6%) | 5 (17,2%) | 0,00 | 0,822 |
| OP AIS Thorax (ohne BWS) | 83 (44,6%) | 24 (82,8%) | 0,07 | <0,001 |
| OP AIS WS (HWS, BWS, LWS) | 32 (17,2%) | 2 (6,9%) | 0,01 | 0,159 |
| OP durchgeführt | 134 (72%) | 26 (89,7%) | 0,02 | 0,043 |
| OP ohne Schädel | 127 (68,3%) | 26 (89,7%) | 0,03 | 0,018 |
| GOS<5 (mind. nicht gut erholt) | 64 (35,2%) | 8 (28,6%) | 0,00 | 0,496 |
| Reha nach Austritt | 73 (39,2%) | 11 (37,9%) | 0,00 | 0,893 |
| Verstorben im Krankenhaus | 24 (12,9%) | 3 (10,3%) | 0,00 | 0,701 |

Die Stärke des statistischen Zusammenhangs und die Signifikanz (t bzw. p) wird durch die Intensität der jeweils verwendeten Farbe veranschaulicht. GCS, Glasgow Coma Scale; (N)ISS, (N)Injury Severity Score; GAP, GCS, Alter und systolischer Blutdruck, RISC2, Revised Injury Severity Classification 2; AIS, Abbreviated Injury Scale;, LEP, LeistungsErfassung von Pflegeleistungen gesamthaft; GOS, Glasgow Outcome Score.

**Suppl. Tabelle D** **Deskriptiver Vergleich aller Patienten mit relevanter Thoraxverletzung (ohne BWS): Ohne vs. mit Thorakotomie /-skopie**

| Thorakotomie/-skopie | Total (N=791) | nein (N=772) | ja (N=19) |  |  |
| --- | --- | --- | --- | --- | --- |
|  | Mean +SD | Mean +SD | Mean +SD | R^2^ | P |
| Alter bei Unfall | 55,5 (+/-20,9) | 55,5 (+/-20,9) | 57,5 (+/-22) | 0,00 | 0,626 |
| 1, syst, Blutdruck | 136,4 (+/-28,9) | 136,5 (+/-28,5) | 131,8 (+/-43,2) | 0,00 | 0,331 |
| 1, Sauerstoffsättigung | 94,4 (+/-7,5) | 94,5 (+/-7,5) | 91,5 (+/-8) | 0,00 | 0,021 |
| 1, GCS | 13,1 (+/-3,6) | 13 (+/-3,6) | 13,4 (+/-2,7) | 0,00 | 0,330 |
| ISS | 19,4 (+/-10,1) | 19,3 (+/-10,1) | 21,3 (+/-10,8) | 0,00 | 0,471 |
| NISS | 23 (+/-11,6) | 22,9 (+/-11,6) | 28,7 (+/-13) | 0,01 | 0,039 |
| GAP | 20,2 (+/-4,2) | 20,2 (+/-4,2) | 19,7 (+/-2,8) | 0,00 | 0,066 |
| RISC2 (%) | 9,43 (+/-20,07) | 9,34 (+/-20,13) | 13,29 (+/-17,7) | 0,00 | 0,002 |
| Age unadjusted Charlson Score | 0,55 (+/-1,19) | 0,53 (+/-1,14) | 1,47 (+/-2,2) | 0,01 | 0,019 |
| AIS1 Kopf/Hals | 1,46 (+/-1,52) | 1,47 (+/-1,52) | 0,84 (+/-1,17) | 0,00 | 0,073 |
| AIS2 Gesicht | 0,31 (+/-0,73) | 0,32 (+/-0,74) | 0 (+/-0) | 0,00 | 0,036 |
| AIS3 Thorax | 2,98 (+/-0,65) | 2,96 (+/-0,63) | 3,84 (+/-0,83) | 0,04 | <0,001 |
| AIS4 Abdomen | 0,78 (+/-1,2) | 0,78 (+/-1,2) | 0,79 (+/-1,23) | 0,00 | 0,935 |
| AIS5 Extremitäten | 1,35 (+/-1,27) | 1,37 (+/-1,27) | 0,63 (+/-1,3) | 0,01 | 0,012 |
| AIS6 Weichteile | 0,45 (+/-0,55) | 0,46 (+/-0,55) | 0,26 (+/-0,45) | 0,00 | 0,128 |
| AIS Schädel/Hirn | 1,37 (+/-1,51) | 1,39 (+/-1,52) | 0,79 (+/-1,13) | 0,00 | 0,099 |
| AIS Thorax (ohne BWS) | 2,96 (+/-0,63) | 2,94 (+/-0,61) | 3,84 (+/-0,83) | 0,05 | <0,001 |
| AIS BWS | 0,49 (+/-0,95) | 0,49 (+/-0,95) | 0,53 (+/-0,91) | 0,00 | 0,748 |
| AIS WS (HWS, BWS, LWS) | 0,84 (+/-1,15) | 0,84 (+/-1,15) | 0,74 (+/-0,99) | 0,00 | 0,806 |
| LEP Total | 6611 (+/-9083) | 6345 (+/-8727) | 17396 (+/-15230) | 0,03 | <0,001 |
| Tage IPS (inkl, 0) | 3,2 (+/-6,5) | 3 (+/-6,2) | 11,2 (+/-12,8) | 0,04 | <0,001 |
| Tage Intubation | 4,9 (+/-6,5) | 4,4 (+/-5,7) | 15,7 (+/-12,4) | 0,13 | 0,003 |
| Hospitalisationstage | 11,6 (+/-12) | 11,4 (+/-11,9) | 21,5 (+/-13,1) | 0,02 | <0,001 |
|  | n (%) | n (%) | n (%) | R^2^ | P |
| Geschlecht weiblich | 203 (25.7%) | 202 (26,2%) | 1 (5,3%) | 0,01 | 0,039 |
| Unfallmechanismus Energie hoch | 527 (66.9%) | 518 (67,4%) | 9 (47,4%) | 0,00 | 0,068 |
| Unfallmechanismus penetrierend | 16 (2%) | 13 (1,7%) | 3 (15,8%) | 0,02 | <0,001 |
| Transport Helikopter | 151 (19.1%) | 148 (19,2%) | 3 (15,8%) | 0,00 | 0,711 |
| Versorgung sekundär | 121 (15.3%) | 117 (15,2%) | 4 (21,1%) | 0,00 | 0,481 |
| ISS ≥16 | 462 (58.4%) | 449 (58,2%) | 13 (68,4%) | 0,00 | 0,371 |
| Mehrfachverletzung | 724 (91.5%) | 712 (92,2%) | 12 (63,2%) | 0,03 | <0,001 |
| Polytrauma (ISS >16 & 2 AIS-Regionen >0) | 455 (57.5%) | 446 (57,8%) | 9 (47,4%) | 0,00 | 0,365 |
| HSM (ISS ≥20 (Kinder ≥16) oder AIS Schädel/Hirn≥3) | 336 (42.5%) | 327 (42,4%) | 9 (47,4%) | 0,00 | 0,663 |
| Maximaler AIS >3 | 206 (26%) | 194 (25,1%) | 12 (63,2%) | 0,02 | <0,001 |
| AIS3 Thorax >2 | 650 (82.2%) | 631 (81,7%) | 19 (100%) | 0,01 | 0,040 |
| AIS4 Abdomen >2 | 72 (9.1%) | 69 (8,9%) | 3 (15,8%) | 0,00 | 0,306 |
| AIS Thorax (ohne BWS) >2 | 647 (81.8%) | 628 (81,3%) | 19 (100%) | 0,01 | 0,037 |
| AIS BWS >2 | 20 (2.5%) | 20 (2,6%) | 0 (0%) | 0,00 | 0,478 |
| AIS WS (HWS, BWS, LWS) >2 | 41 (5.2%) | 41 (5,3%) | 0 (0%) | 0,00 | 0,303 |
| Klin, Notfallintervention (Not-Op, DGU) | 80 (10.1%) | 76 (9,8%) | 4 (21,1%) | 0,00 | ,110 |
| Chirurg, Not-Eingriff inkl, Präklinik (Tx,drain,, Not-OP) | 184 (23.3%) | 169 (21,9%) | 15 (78,9%) | 0,04 | <,001 |
| Chirurg, Not-Eingriff inkl, Präklinik (Tx,drain,, Not-OP) oder Damage Control | 217 (27.4%) | 202 (26,2%) | 15 (78,9%) | 0,03 | <,001 |
| Thoraxdrainage (Präklinik oder SR) | 128 (16.2%) | 114 (14,8%) | 14 (73,7%) | 0,06 | <,001 |
| Blut- oder Hämostasetherapie SR/IPS erhalten | 210 (26.5%) | 200 (25,9%) | 10 (52,6%) | 0,01 | ,009 |
| DGU IPS Aufenthalt (aus Zeiten) | 381 (48.2%) | 365 (47,3%) | 16 (84,2%) | 0,01 | ,001 |
| Intubiert Präklinik oder SR | 160 (20.2%) | 153 (19,8%) | 7 (36,8%) | 0,00 | ,068 |
| Intubiert (Präklinik bis IPS, ohne OP) | 241 (30.5%) | 232 (30,1%) | 9 (47,4%) | 0,00 | ,105 |
| OP AIS3 Thorax | 144 (18.2%) | 127 (16,5%) | 17 (89,5%) | 0,08 | <,001 |
| OP AIS4 Abdomen | 67 (8.5%) | 66 (8,5%) | 1 (5,3%) | 0,00 | ,612 |
| OP AIS Thorax (ohne BWS) | 107 (13.5%) | 90 (11,7%) | 17 (89,5%) | 0,12 | <,001 |
| OP AIS WS (HWS, BWS, LWS) | 77 (9.7%) | 75 (9,7%) | 2 (10,5%) | 0,00 | ,906 |
| OP durchgeführt | 388 (49.1%) | 371 (48,1%) | 17 (89,5%) | 0,02 | <,001 |
| OP ohne Schädel | 355 (44.9%) | 338 (43,8%) | 17 (89,5%) | 0,02 | <,001 |
| GOS<5 (mind, nicht gut erholt) | 219 (28.2%) | 214 (28,2%) | 5 (27,8%) | 0,00 | ,969 |
| Reha nach Austritt | 219 (27.7%) | 210 (27,2%) | 9 (47,4%) | 0,00 | ,052 |
| Verstorben im Krankenhaus | 85 (10.7%) | 83 (10,8%) | 2 (10,5%) | 0,00 | ,975 |

Die Stärke des statistischen Zusammenhangs und die Signifikanz (t bzw. p) wird durch die Intensität der jeweils verwendeten Farbe veranschaulicht. GCS, Glasgow Coma Scale; (N)ISS, (N)Injury Severity Score; GAP, GCS, Alter und systolischer Blutdruck, RISC2, Revised Injury Severity Classification 2; AIS, Abbreviated Injury Scale;, LEP, LeistungsErfassung von Pflegeleistungen gesamthaft; GOS, Glasgow Outcome Score.

**Suppl. Tabelle E Logistische Regression (block- und schrittweise) bzgl. Notwendigkeit einer Thorakotomie/ -skopie aller relevant Thoraxverletzten (19 von 791)**

| Block | Schritt | Variablen | B | p | OR | 95% CI | |  | Gesamt | |  | Verbesserung | |
| --- | --- | --- | --- | --- | --- | --- | --- | --- | --- | --- | --- | --- | --- |
|  |  |  |  |  |  | unterer | oberer |  | *p* | R^2^ |  | *p* | R^2^ |
| Demografie und Unfall | 1 | Penetrierend | 2,76 | 0,001 | 15,78 | 2,88 | 86,50 |  | <0,001 | 0,17 |  |  |  |
|  | 2 | Charlson Score | 0,36 | 0,010 | 1,44 | 1,09 | 1,89 |  | <0,001 | 0,22 |  | 0,004 | 0,05 |
| Verletzung | 3 | AIS Thorax (ohne BWS) | 1,58 | <0,001 | 4,87 | 2,50 | 9,48 |  | <0,001 | 0,27 |  | 0,005 | 0,05 |
|  | 4 | AIS Extremitäten | -0,84 | 0,001 | 0,43 | 0,26 | 0,72 |  | <0,001 | 0,31 |  | 0,006 | 0,05 |
| Versorgung | 5 | LEP Total | <0,00005 | <0,001 | 1,00005 | 1,00001 | 1,00009 |  | <0,001 | 0,39 |  | <0,001 | 0,08 |
|  |  | Constant | -9,51 | <0,001 | <0,001 |  |  |  |  |  |  |  |  |

Gesamt: Gesamtmodell; Verbesserung: Verbesserung des Modells pro Schritt; B: Regressionskoeffizient B; OR (Odds Ratio) = EXP (B); 95% CI 95% (Konfidenzintervall); R^2^: Nagelkerke R^2^

AIS, Abbreviated Injury Scale; LEP, Leistungserfassung von Pflegeleistungen.

**Suppl. Tabelle F Logistische Regression (block- und schrittweise) bzgl. Notwendigkeit einer Thorakotomie/ -skopie unter allen Patienten mit erfolgtem Thoraxeingriff (19 von 215)**

| Block | Schritt | Variablen | B | p | OR | 95% CI | |  | Gesamt | |  | Verbesserung | |
| --- | --- | --- | --- | --- | --- | --- | --- | --- | --- | --- | --- | --- | --- |
|  |  |  |  |  |  | unterer | oberer |  | *P* | R^2^ |  | *p* | R^2^ |
| Demografie und Unfall | 1 | Charlson Score | 0,49 | 0,011 | 1,625 | 1,117 | 2,366 |  | 0,002 | 0,10 |  |  |  |
|  | 2 | Penetrierend | 1,69 | 0,047 | 5,397 | 1,020 | 28,539 |  | 0,001 | 0,14 |  | 0,039 | 0,04 |
| Verletzung | 3 | AIS Thorax (ohne BWS) | 1,02 | 0,003 | 2,786 | 1,423 | 5,454 |  | <0,001 | 0,22 |  | 0,003 | 0,09 |
|  | 4 | AIS Extremitäten | -0,83 | 0,001 | 0,435 | 0,262 | 0,722 |  | <0,001 | 0,32 |  | 0,001 | 0,09 |
| Versorgung | 5 | LEP Total | <0,00005 | <0,001 | 1,00005 | 1,00001 | 1,00009 |  | <0,001 | 0,37 |  | 0,014 | 0,05 |
|  |  | Constant | 6,30 | <0,001 | 0,002 |  |  |  |  |  |  |  |  |

Gesamt: Gesamtmodell; Verbesserung: Verbesserung des Modells pro Schritt; B: Regressionskoeffizient B; OR (Odds Ratio) = EXP (B); 95% CI 95% (Konfidenzintervall); R^2^: Nagelkerke R^2^

AIS, Abbreviated Injury Scale; LEP, Leistungserfassung von Pflegeleistungen.

**Suppl. Tabelle G** **Deskriptiver Vergleich aller mittels Thorakoskopie oder –tomie versorgten Patienten bzgl. notfallmässig oder nicht notfallmässig durchgeführtem Eingriff**

| Thorakaler Notfalleingriff (innert 24h) | Total (N=19) | >24h (N=11) | innert 24h (N=8) |  |  |
| --- | --- | --- | --- | --- | --- |
|  | Mean +SD | Mean +SD | Mean +SD | R^2^ | P |
| Alter bei Unfall | 57,5 (+/-21,9) | 63,6 (+/-17,6) | 49,1 (+/-25,6) | 0,11 | 0,283 |
| 1, syst, Blutdruck | 131,8 (+/-43,1) | 138,1 (+/-48,9) | 123,3 (+/-34,9) | 0,03 | 0,620 |
| 1, Sauerstoffsättigung | 91,5 (+/-8) | 91,7 (+/-9,3) | 91,1 (+/-6,2) | 0,00 | 0,507 |
| 1, GCS | 13,4 (+/-2,7) | 13,9 (+/-1,5) | 12,8 (+/-3,7) | 0,05 | 0,607 |
| ISS | 21,3 (+/-10,8) | 16 (+/-6,6) | 28,6 (+/-11,6) | 0,35 | 0,020 |
| NISS | 28,7 (+/-13) | 22,4 (+/-9,3) | 37,4 (+/-12,7) | 0,34 | 0,016 |
| GAP | 19,7 (+/-2,8) | 20,3 (+/-1,5) | 19 (+/-3,9) | 0,06 | 0,820 |
| RISC2 (%) | 13,29 (+/-17,7) | 6,68 (+/-6,26) | 22,37 (+/-24,22) | 0,20 | 0,126 |
| Age unadjusted Charlson Score | 1,47 (+/-2,2) | 1,55 (+/-1,81) | 1,38 (+/-2,77) | 0,00 | 0,474 |
| AIS1 Kopf/Hals | 0,84 (+/-1,17) | 1 (+/-1,34) | 0,63 (+/-0,92) | 0,03 | 0,609 |
| AIS2 Gesicht | 0 (+/-0) | 0 (+/-0) | 0 (+/-0) |  |  |
| AIS3 Thorax | 3,84 (+/-0,83) | 3,36 (+/-0,67) | 4,5 (+/-0,54) | 0,48 | 0,003 |
| AIS4 Abdomen | 0,79 (+/-1,23) | 0,45 (+/-1,04) | 1,25 (+/-1,39) | 0,11 | 0,159 |
| AIS5 Extremitäten | 0,63 (+/-1,3) | 0,18 (+/-0,6) | 1,25 (+/-1,75) | 0,17 | 0,105 |
| AIS6 Weichteile | 0,26 (+/-0,45) | 0,09 (+/-0,3) | 0,5 (+/-0,54) | 0,21 | 0,052 |
| AIS Schädel/Hirn | 0,79 (+/-1,13) | 1 (+/-1,34) | 0,5 (+/-0,76) | 0,05 | 0,487 |
| AIS Thorax (ohne BWS) | 3,84 (+/-0,83) | 3,36 (+/-0,67) | 4,5 (+/-0,54) | 0,48 | 0,003 |
| AIS BWS | 0,53 (+/-0,91) | 0,36 (+/-0,81) | 0,75 (+/-1,04) | 0,05 | 0,358 |
| AIS WS (HWS, BWS, LWS) | 0,74 (+/-0,99) | 0,36 (+/-0,81) | 1,25 (+/-1,04) | 0,21 | 0,054 |
| LEP Total | 17396 (+/-15230) | 14729 (+/-16041) | 21063 (+/-14225) | 0,04 | 0,248 |
| Tage IPS (inkl, 0) | 11,2 (+/-12,8) | 9,5 (+/-14,5) | 13,6 (+/-10,3) | 0,03 | 0,160 |
| Tage Intubation | 15,7 (+/-12,4) | 25,4 (+/-11,8) | 10,8 (+/-10,3) | 0,34 | 0,121 |
| Hospitalisationstage | 21,5 (+/-13,1) | 19,3 (+/-13,4) | 24,6 (+/-12,8) | 0,04 | 0,457 |
|  | n (%) | n (%) | n (%) | R^2^ | P |
| Geschlecht weiblich | 1 (5.3%) | 1 (9,1%) | 0 (0%) | 0,04 | 0,409 |
| Unfallmechanismus Energie hoch | 9 (47.4%) | 4 (36,4%) | 5 (62,5%) | 0,07 | 0,285 |
| Unfallmechanismus penetrierend | 3 (15.8%) | 1 (9,1%) | 2 (25%) | 0,05 | 0,376 |
| Transport Helikopter | 3 (15.8%) | 1 (9,1%) | 2 (25%) | 0,05 | 0,376 |
| Versorgung sekundär | 4 (21.1%) | 2 (18,2%) | 2 (25%) | 0,01 | 0,737 |
| ISS ≥16 | 13 (68.4%) | 5 (45,5%) | 8 (100%) | 0,34 | 0,009 |
| Mehrfachverletzung | 12 (63.2%) | 7 (63,6%) | 5 (62,5%) | 0,00 | 0,962 |
| Polytrauma (ISS >16 & 2 AIS-Regionen >0) | 9 (47.4%) | 4 (36,4%) | 5 (62,5%) | 0,07 | 0,285 |
| HSM (ISS ≥20 (Kinder ≥16) oder AIS Schädel/Hirn≥3) | 9 (47.4%) | 4 (36,4%) | 5 (62,5%) | 0,07 | 0,285 |
| Maximaler AIS >3 | 12 (63.2%) | 4 (36,4%) | 8 (100%) | 0,42 | 0,003 |
| AIS3 Thorax >2 | 19 (100%) | 11 (100%) | 8 (100%) |  |  |
| AIS4 Abdomen >2 | 3 (15.8%) | 1 (9,1%) | 2 (25%) | 0,05 | 0,376 |
| AIS Thorax (ohne BWS) >2 | 19 (100%) | 11 (100%) | 8 (100%) |  |  |
| AIS BWS >2 | 0 (0%) | 0 (0%) | 0 (0%) |  |  |
| AIS WS (HWS, BWS, LWS) >2 | 0 (0%) | 0 (0%) | 0 (0%) |  |  |
| Klin, Notfallintervention (Not-Op, DGU) | 4 (21.1%) | 0 (0%) | 4 (50%) | 0,37 | 0,006 |
| Chirurg, Not-Eingriff inkl, Präklinik (Tx,drain,, Not-OP) | 15 (78.9%) | 8 (72,7%) | 7 (87,5%) | 0,03 | 0,464 |
| Chirurg, Not-Eingriff inkl, Präklinik (Tx,drain,, Not-OP) oder Damage Control | 15 (78.9%) | 8 (72,7%) | 7 (87,5%) | 0,03 | 0,464 |
| Thoraxdrainage (Präklinik oder SR) | 14 (73.7%) | 8 (72,7%) | 6 (75%) | 0,00 | 0,918 |
| Blut- oder Hämostasetherapie SR/IPS erhalten | 10 (52.6%) | 4 (36,4%) | 6 (75%) | 0,15 | 0,106 |
| DGU IPS Aufenthalt (aus Zeiten) | 16 (84.2%) | 8 (72,7%) | 8 (100%) | 0,14 | 0,120 |
| Intubiert Präklinik oder SR | 7 (36.8%) | 2 (18,2%) | 5 (62,5%) | 0,21 | 0,051 |
| Intubiert (Präklinik bis IPS, ohne OP) | 9 (47.4%) | 3 (27,3%) | 6 (75%) | 0,22 | 0,041 |
| OP AIS3 Thorax | 17 (89.5%) | 9 (81,8%) | 8 (100%) | 0,09 | 0,224 |
| OP AIS4 Abdomen | 1 (5.3%) | 1 (9,1%) | 0 (0%) | 0,04 | 0,409 |
| OP AIS Thorax (ohne BWS) | 17 (89.5%) | 9 (81,8%) | 8 (100%) | 0,09 | 0,224 |
| OP AIS WS (HWS, BWS, LWS) | 2 (10.5%) | 1 (9,1%) | 1 (12,5%) | 0,00 | 0,824 |
| OP durchgeführt | 17 (89.5%) | 9 (81,8%) | 8 (100%) | 0,09 | 0,224 |
| OP ohne Schädel | 17 (89.5%) | 9 (81,8%) | 8 (100%) | 0,09 | 0,224 |
| GOS<5 (mind, nicht gut erholt) | 5 (27.8%) | 3 (27,3%) | 2 (28,6%) | 0,00 | 0,956 |
| Reha nach Austritt | 9 (47.4%) | 5 (45,5%) | 4 (50%) | 0,00 | 0,855 |
| Verstorben im Krankenhaus | 2 (10.5%) | 1 (9,1%) | 1 (12,5%) | 0,00 | 0,824 |

Die Stärke des statistischen Zusammenhangs und die Signifikanz (t bzw. p) wird durch die Intensität der jeweils verwendeten Farbe veranschaulicht. GCS, Glasgow Coma Scale; (N)ISS, (N)Injury Severity Score; GAP, GCS, Alter und systolischer Blutdruck, RISC2, Revised Injury Severity Classification 2; AIS, Abbreviated Injury Scale;, LEP, LeistungsErfassung von Pflegeleistungen gesamthaft; GOS, Glasgow Outcome Score.
